# Supplementary figures and images for: Buildup and bistability in auditory streaming as an evidence accumulation process with saturation
Source: PLoS Comput Biol. 2020 Aug 27;16(8):e1008152. doi: 10.1371/journal.pcbi.1008152 (PMC7480857; doi:10.1371/journal.pcbi.1008152)

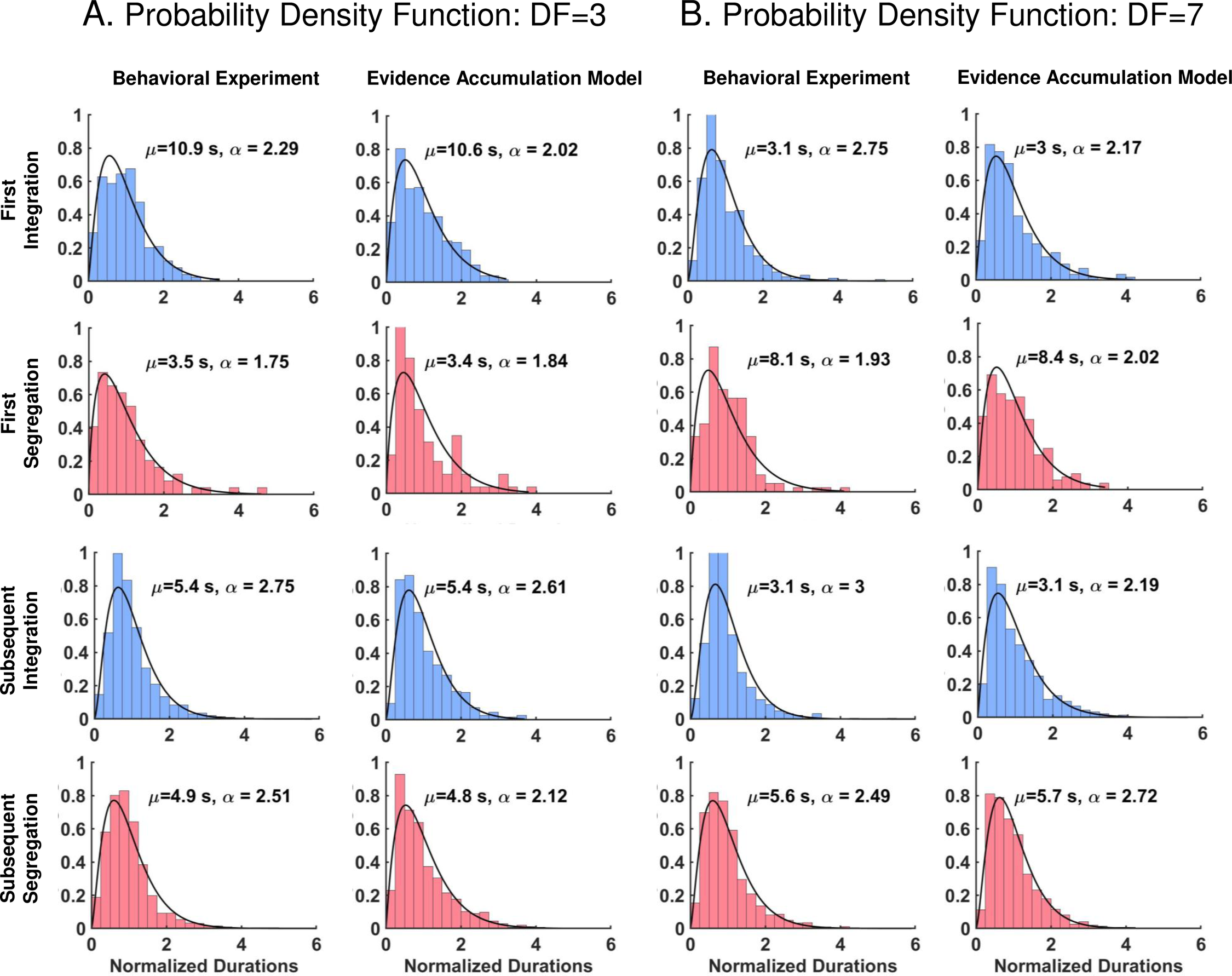

Supplement: S1 Fig — Distributions of normalized phase durations are shown for A: DF = 3 and B: DF = 7. They are obtained from numerical simulations of the EVA model (columns 2,4) and compared to those derived from behavioral data (columns 1,3). (TIF) [file pcbi.1008152.s001.tif]

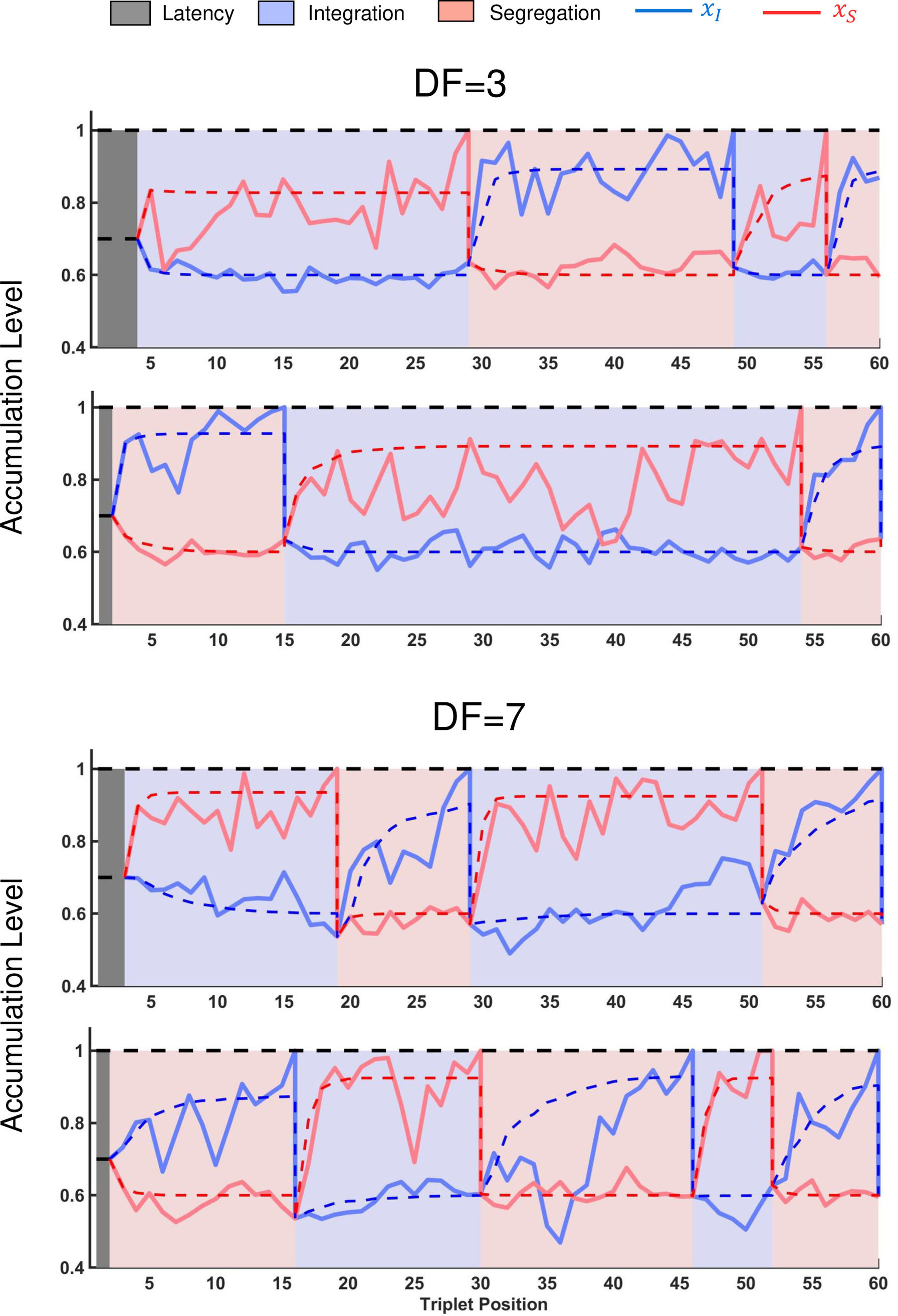

Supplement: S2 Fig — In only a few trials, 103 out of 675 for DF = 3 and 220 out of 675 for DF = 7, the first percept is segregation (see panels 2,4). During a cycle, the suppressed unit accumulates evidence against the current percept until it reaches the switching threshold. Then, a perceptual switch occurs and accumulators are reset to the same value. In the noise free case, the accumulators stabilize to their corresponding target values and there are no alternations. Such trajectories are depicted by dashed lines. (TIF) [file pcbi.1008152.s002.tif]

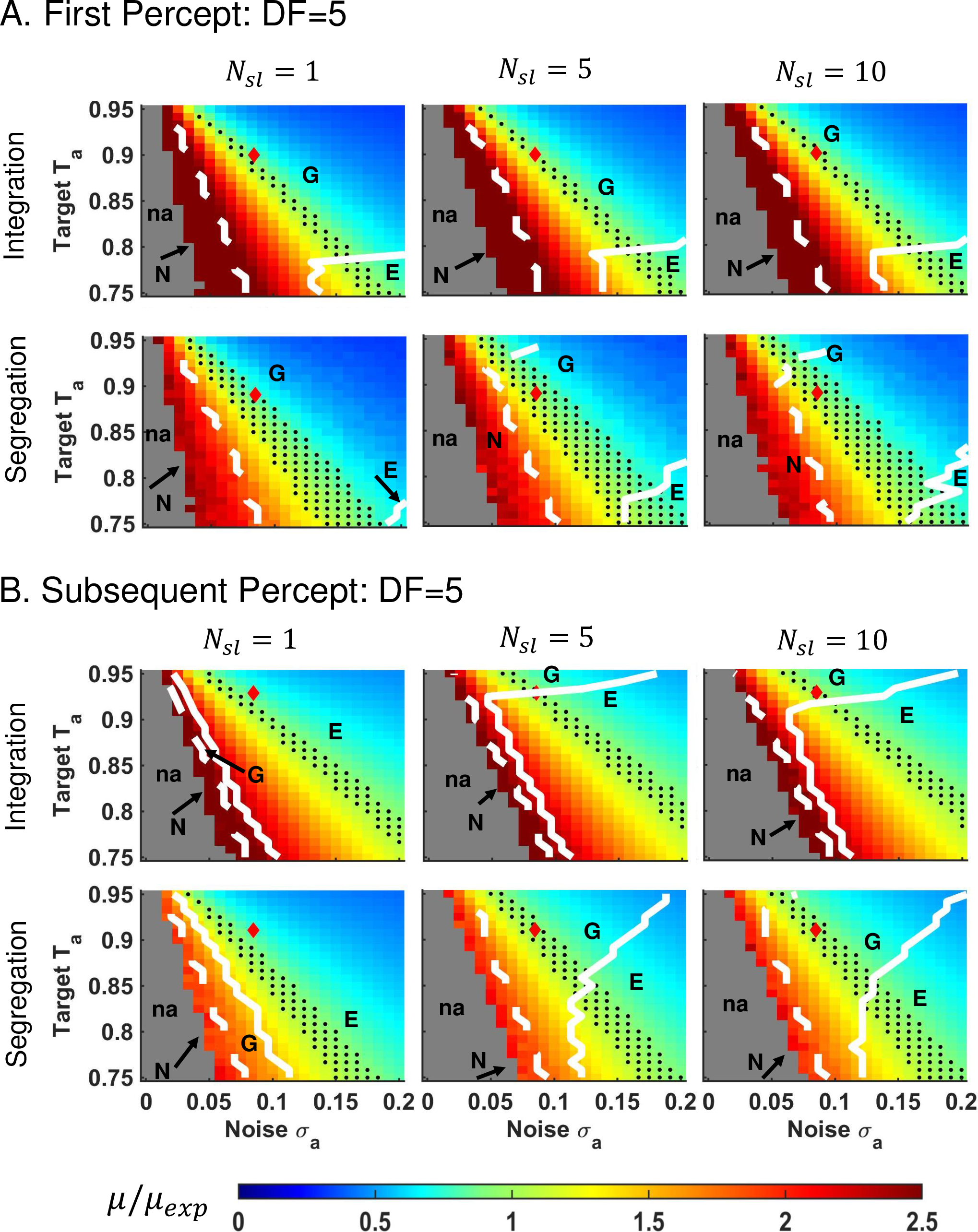

Supplement: S3 Fig — Some important differences occur, however, at Nsl = 1 (e.g. for subsequent durations). A two-parameter response diagram of the dependence of μ and α on target-against Ta and noise strength σa is shown for DF = 5 and varying Nsl for A: first percepts and B: subsequent percepts. All parameters are chosen as described in Methods, except for Nsl (here Nsl = 1, 5 or 10). For comparison, see Fig 7, middle column; Nsl = 20 at DF = 5. Red diamonds correspond to same parameter choices as in Fig 7 for Nsl = 20, as well. The heat map represents the ratio μ/μexp between model-generated μ and mean duration μexp from the behavioral data. Regions of no alternations (na) are colored in gray. Mean durations are much longer than their experimental counterparts μexp (region in warm colors), much shorter than μexp (region in cool colors), or close to μexp (within one standard error to μexp; in green; black dots depict a discrete selection of values in the green region). The distributions of normalized percept durations are characterized by three distinct regions: for small σa the distributions are normal (region N, to the left of dashed-white line; α ≫ 3); for large σa the distributions are exponential (region E, to the right of solid-white line; α near 1); for intermediate values σa the distributions are gamma-like with shape close to that found experimentally (region G, between white contours; α ≈ 2 for first percepts and α ≈ 2.6 for subsequent percepts; α differs from αexp by relative error up to 20% except for integration at DF = 7 where it is up to 30%). As in Fig 7, middle column, the intersection of white contours with the sheet of black dots identifies parameter values that yield well-fit data. Note that at Nsl = 1 this intersection is empty for both subsequent percepts I and S (panel B, first column). (TIF) [file pcbi.1008152.s003.tif]

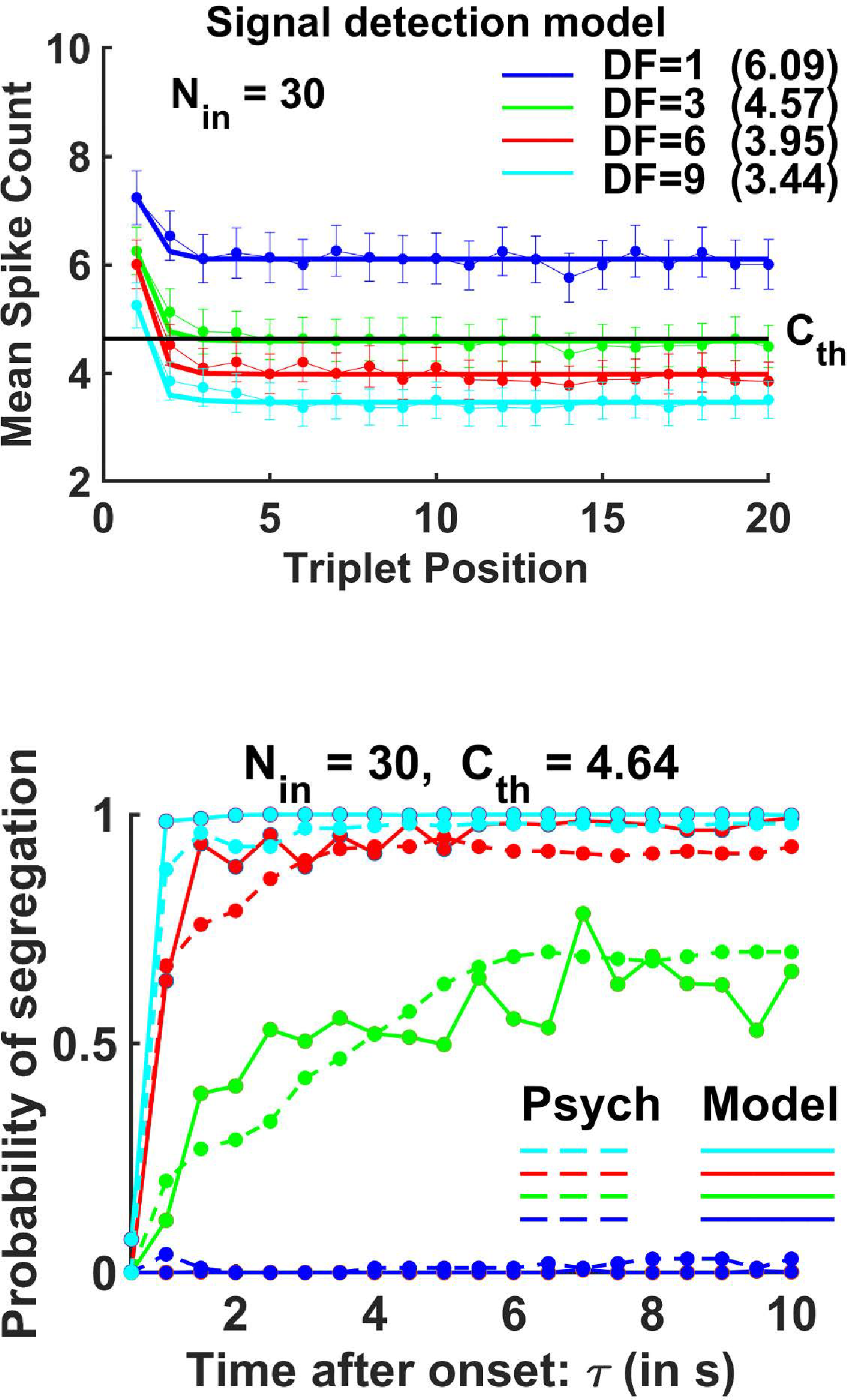

Supplement: S4 Fig — For comparison, see Micheyl et al (2005) [1]. Upper panel: mean spike counts mt;DF (scatter points) at A-tone selective neurons in A1 during tone B were extracted from [1, (Fig.3A in Ref)]. They correspond to conditions DF = 1 (blue), 3 (green), 6 (red), 9 (cyan), based on 10 s (20 triplets) long trials. The mean spike counts decrease exponentially and stabilize within a few seconds (solid curves for the exponential fits). The algorithm generates spike counts during B-tone by using Poisson processes of means mt;DF, and then average them over Nin neuronal units. The average values of the mean spike counts, including asymptotic values (written in parenthesis) at each DF, and the standard error to the mean (SEM) are computed over 675 trials. Lower panel: The signal detection algorithm constructs neurometric functions using numerical data from all Nin neuronal units. Parameters Nin and Cth are chosen to yield SEM similar to those observed in the spike count data [1, (Fig.3A in Ref)] and to yield the least-squares error of the experimental buildups (dashed, extracted from [1, (Fig.4 in Ref)] and the computer-simulated neurometric functions (solid) for DF = 1, 3, 6, 9. The best approximation is obtained for Nin = 30, Cth = 4.64. Note: Statistics of percept durations were not reported in [1]; this prevented us from comparing these aspects of behavioral data from [1] to our numerically-generated duration distributions at DF = 1, 3, 6, 9. (TIF) [file pcbi.1008152.s004.tif]

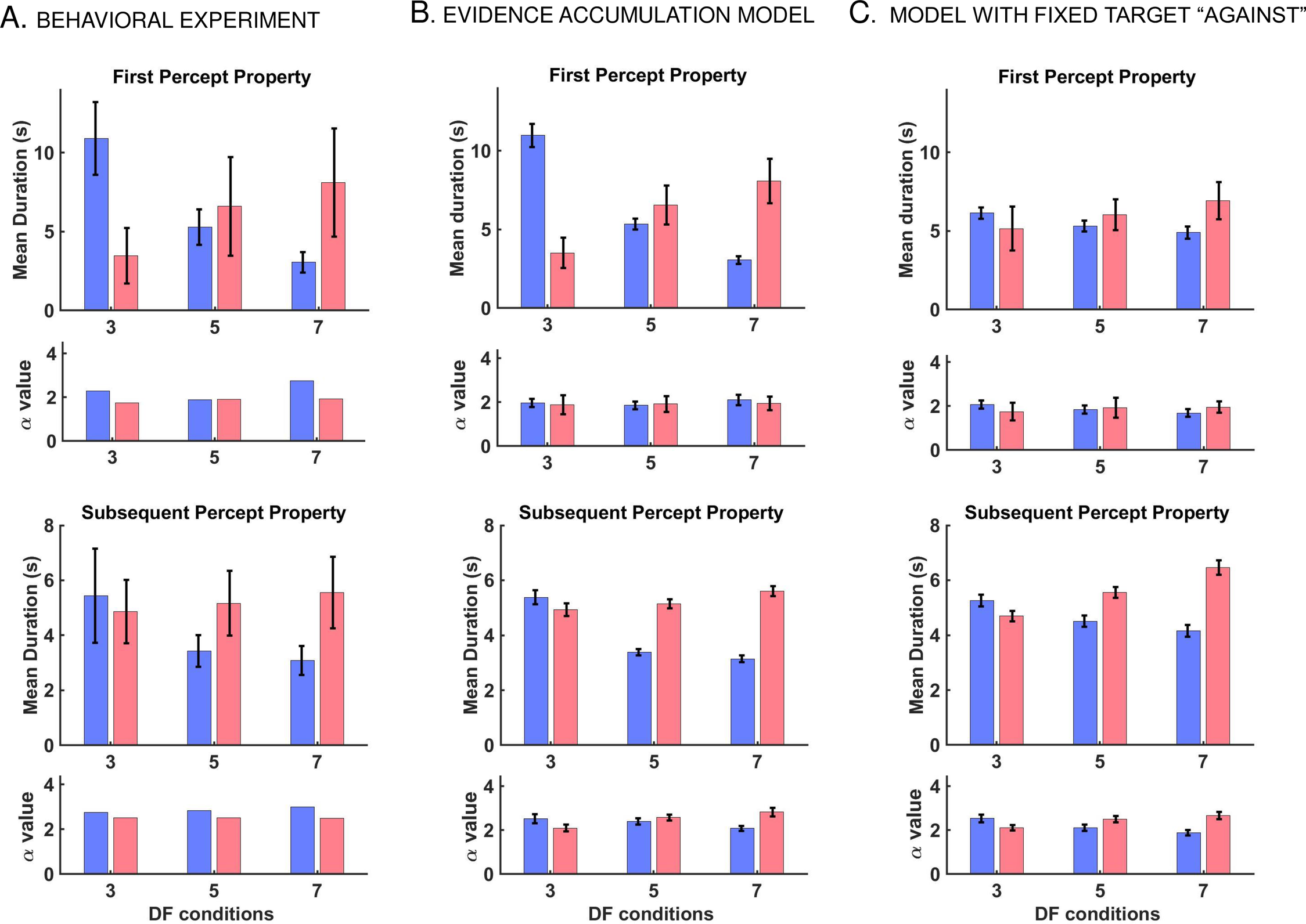

Supplement: S5 Fig — For comparison, mean durations and shape parameter α of gamma distributions are shown for A: Experimental data; B: EVA model with optimized values for target-against (see Methods, Parameter values used in model simulations). EVA-generated results are identical to those in Figs 5 and 6; and C: Non-optimized EVA simulated with Ta = 0.9 across all DF = 3, 5, 7 and first and subsequent I, S. All other parameters are as in panel B. The mean durations from simulations follow the trend of experimental data which is decreasing/increasing with DF for I/S respectively. However, they fail to approximate well the entire set of behavioral data (e.g. approximations of mean first durations at DF = 3 and DF = 7 are inaccurate). On the other hand, gamma-fit shape values α are comparable to those from panels A and B. This is not surprising given that α depends mostly on the noise-level σa, as shown in Fig 7. (TIF) [file pcbi.1008152.s005.tif]
